# Aortic Valve Leaflet Shape Synthesis With Geometric Prior From Surrounding Tissue

**DOI:** 10.3389/fcvm.2022.772222

**Published:** 2022-03-09

**Authors:** Jannis Hagenah, Michael Scharfschwerdt, Floris Ernst

**Affiliations:** ^1^Institute for Robotics and Cognitive Systems, University of Lübeck, Lübeck, Germany; ^2^Department of Engineering Science, University of Oxford, Oxford, United Kingdom; ^3^Department of Cardiac Surgery, University-Hospital Schleswig-Holstein, Lübeck, Germany

**Keywords:** deep learning, shape synthesis, representation learning, domain gap, aortic valve

## Abstract

Even though the field of medical imaging advances, there are structures in the human body that are barely assessible with classical image acquisition modalities. One example are the three leaflets of the aortic valve due to their thin structure and high movement. However, with an increasing accuracy of biomechanical simulation, for example of the heart function, and extense computing capabilities available, concise knowledge of the individual morphology of these structures could have a high impact on personalized therapy and intervention planning as well as on clinical research. Thus, there is a high demand to estimate the individual shape of inassessible structures given only information on the geometry of the surrounding tissue. This leads to a domain adaptation problem, where the domain gap could be very large while typically only small datasets are available. Hence, classical approaches for domain adaptation are not capable of providing sufficient predictions. In this work, we present a new framework for bridging this domain gap in the scope of estimating anatomical shapes based on the surrounding tissue's morphology. Thus, we propose deep representation learning to not map from one image to another but to predict a latent shape representation. We formalize this framework and present two different approaches to solve the given problem. Furthermore, we perform a proof-of-concept study for estimating the individual shape of the aortic valve leaflets based on a volumetric ultrasound image of the aortic root. Therefore, we collect an *ex-vivo* porcine data set consisting of both, ultrasound volume images as well as high-resolution leaflet images, evaluate both approaches on it and perform an analysis of the model's hyperparameters. Our results show that using deep representation learning and domain mapping between the identified latent spaces, a robust prediction of the unknown leaflet shape only based on surrounding tissue information is possible, even in limited data scenarios. The concept can be applied to a wide range of modeling tasks, not only in the scope of heart modeling but also for all kinds of inassessible structures within the human body.

## 1. Introduction

Despite ongoing advancements of medical imaging techniques, there are structures in the human body that are difficult to visualize using typical medical imaging modalities like Computed Tomography (CT), Magnetic Resonance Imaging (MRI), or Ultrasound Imaging (US). However, the knowledge of these structures' shape is highly relevant for clinical decision making and intervention, e.g., for biomechanical simulations or for personalized prosthesis shaping. One example of such a structure is the aortic valve. This valve consists of three thin leaflets embedded in the aortic root that are pressed together during diastole to prevent the blood in the aorta from flowing back into the left ventricle (1). The geometry of the aortic valve, i.e., the shape of its three leaflets, shows an enormous inter-patient variability (2). As the aortic valve and root form a complex biomechanical system with a lot of interaction between different anatomical structures (3), this individual geometry is crucial for the correct functionality of the valve. The valve's leaflets are extremely thin and flutter in the blood stream, hence, imaging them using typical medical imaging modalities is challenging. However, the knowledge about the individual geometry of the aortic valve is necessary for many applications, ranging from heart modeling and simulation to the development of personalized prostheses. For both applications, the leaflet shape should be assessed in an unpressurized state to avoid stress-related deformations. Due to the high impact of the individual valve geometry on a procedure's outcome, a personalization of this prosthesis would be beneficial but is out of scope as the desired prosthesis shape is not assessible using typical *in-vivo* imaging modalities. Hence, the synthesis of personalized leaflet shapes presents a promising approach for solving this problem.

In an *ex-vivo* setting, it is possible to extract the aortic valve leaflets and collect high-resolution images of them in a planar state (4). Such a dataset could serve as a training set for a generative model aiming on leaflet shape synthesis. An additional advantage of this approach is that the synthesized leaflet shapes are already in a planar shape, allowing for directly manufacturing a prosthesis out of typical material, e.g., pericardium. However, the shape synthesis should be tailored to the individual patient. Thus, the generative model should receive a prior in the form of information of relevant surrounding tissue to estimate the patient's individual leaflet shapes. In this case, the three-dimensional (3D) shape of the aortic root might be a sufficient prior as its geometry should be closely related to the shape of the individual leaflet. Additionally, the aortic root is clearly visible in ultrasound images acquired using transesophageal echocardiography (TEE).

However, the domain gap between a 3D ultrasound image of the aortic root where the leaflets are barely visible and an RGB image of the leaflet shape in its planar state is huge. The image's appearance highly differs between both modalities and even though the acquired organ is the same, both modalities assess very different parts of the organ with only little overlap across the modalities. Typical methods for bridging domain gaps utilize adversarial training, i.e., generative adversarial networks (GAN), to transfer an image from one domain to the other one (5). However, GANs need vast amounts of training data to converge that is typically not available in medical imaging. Especially for structure shape synthesis based on surrounding tissue, data collection is very time-consuming and requires extra effort due to *ex-vivo* experiments. Additionally, GANs are prone to sometimes synthesizing unrealistic images (6), which should be avoided in the scope of medical decision support systems or prosthesis manufacturing.

In this work, we present a robust approach for synthesizing aortic valve leaflet shapes with the individual aortic root shape as geometric prior based on shape encoding with autoencoders. First, we collect and present a dataset containing 3D US images of porcine aortic roots as well as 2D planar images of the corresponding leaflets. Then, we formalize the given problem and present different ways to solve it. We evaluate all these approaches on the collected dataset, including a hyperparameter analysis and a comparison of the different approaches.

### 1.1. Contribution of This Work

The contribution of this work is three-fold. First, to the best of our knowledge, we present the first sufficiently large dataset containing 3D aortic root shapes as well as high resolution images of the corresponding valve leaflets. Second, we describe a novel methodology for bridging big domain gaps that works robustly even on small data. As this method is not limited to aortic valve leaflet synthesis, our methodological contribution might be of great interest in the medical image analysis community as well as for general computer vision researchers. Third, our proposed method can be directly applied in the scope of personalized aortic valve modeling, for example for prosthesis development or cardiac simulation, highlighting not only the methodological contribution but also the clinical applicability and translational value of this study.

## 2. Related Work

In general, dealing with data from different domains is referred to as domain adaptation (7). Lots of studies aimed at generalizing across different domains (8–10). However, the typical focus is dealing with input data from different domains, not to transfer an image to another domain (11).

The problem of estimating an image based on another image is called image-to-image translation and was introduced by (5). Typically, generative adversarial networks (GAN) (12) are utilized to learn a distribution over the target images, conditioned by the input image (5). Such approaches have been applied to a wide range of topics, for example semantic image synthesis (13), image segmentation (14), style transfer (15), or image inpainting (16). However, most of these approaches are used in settings with a moderate domain gap while the general objects in the input image remain unchanged in their shape.

Several studies focused on image-to-image-translation for shape deformation, aiming on bridging a wider domain gap (17). However, all these approaches were evaluated on large datasets that are typically not available in clinical applications. Even though, Liu et al. (18) proposed a few-shot approach for image-to-image translation, the model has to be pre-trained on a large dataset that is relatively similar to the small one. Lin et al. (19) present a framework based on a cascaded GAN structure to learn image-to-image translation from one image pair only. However, such a model is not capable of generating the full variance of anatomies present across patients as it is trained to fit one anatomy as closely as possible. In contrast, our approach directly models this variance by encoding all observed anatomies into latent space descriptions.

Several studies aimed at personalized modeling of the aortic valve leaflet shape. Typically, this is either done by segmenting the leaflets from CT data (20) or by deforming a template leaflet shape according to the surrounding tissue anatomy extracted from a CT (21). Both approaches incorporate a substantial amount of expert knowledge as well as manual interaction. Additionally, due to the deformation constraints, they are biased toward predicting an average leaflet shape.

In a previous study, it could be shown that the aortic root shape carries enough information to estimate specific features of the valve leaflets, i.e., the commissure contour line (22). Thus, a support vector regression model was trained based on manually identified geometric features. Even though the commissure line was predictable, the model was not capable of estimating the full leaflet shape, i.e., synthesizing images of the leaflets. Additionally, the evaluation was performed on a small dataset and the ultrasound volumes were stitched together and interpolated from several rotated 2D imaging planes. The approach to use autoencoders to derive a sufficient latent space containing the shape of aortic valve leaflets was presented in Hagenah et al. (23). However, this study focused on shape typification in latent space and no connection between the leaflet shape and the aortic root geometry was identified.

## 3. Materials and Methods

In this chapter, our methodology is presented. First, we present the data collection procedure and the preprocessing applied to the raw data (section 3.1). Then, we formalize the problem of domain mapping for leaflet shape synthesis and present general approaches to solve it (section 3.2). In sections 3.3 and 3.4, our proposed methods for solving the problem are presented. As there is a wide variety of interpretations of the term *shape*, it is important to note that we interpret the shape of a leaflet as its segmentation in a 2D image that shows the leaflet in a spreaded state.

### 3.1. Data Collection

One big challenge of collecting a sufficiently large dataset is that acquiring high-resolution images of the aortic valve leaflets is barely possible *in-vivo*. One typical method is to extract the single leaflets and photograph them in a planar shape (4, 23). Hence, we followed an *ex-vivo* approach examining porcine hearts. The pig heart's anatomy and physiology are quite similar to the human one making it a common animal model (24). This also holds for the aortic valve apparatus as porcine valves are even used as xenograft prostheses (25). As the pig hearts were bought at a slaughterhouse, there are no ethical concerns raised by this study. All in all, we collected data of 29 porcine hearts. From each of these hearts, we extracted and acquired a volumetric ultrasound image of the aortic root, followed by an extraction of the leaflets and their image acquisition. Therefore, the collected data consists of 29 ultrasound volumes and photographs of the right-coronary, left-coronary, and non-coronary leaflet, respectively, so 87 leaflet images in total. Details on the setups, the workflow and data preprocessing are described in the following paragraphs.

#### 3.1.1. Aortic Root Imaging

**Figure 1:**
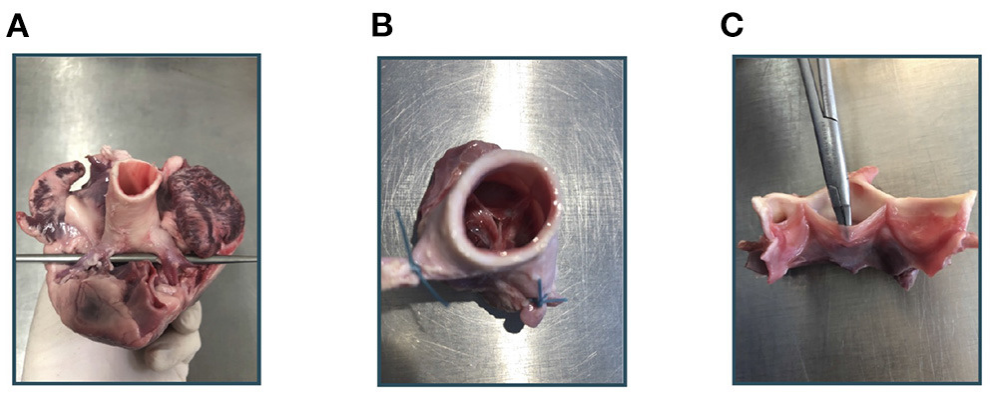
Experimental workflow. At first, the aortic root is extracted from the porcine heart **(A)**. After clamping the coronary arteries **(B)**, an ultrasound image of the aortic root can be acquired. Then, the root is cut vertically and the leaflets can be extracted **(C)**.

Imaging of the aortic root mainly followed the method described in Hagenah et al. (26). From the fresh pig heart, the aortic root was extracted by exposing the aorta and then cutting the root out of the left ventricle around the ventriculoarterial junction. The coronary arteries were clamped, the root was attached to a vertical tube within a water basin and, using a water column within this tube, a constant, physiologically realistic diastolic pressure was applied. Hence, the valve appeared in a closed state. Figure 1 shows photographs of the extraction steps. Within the water basin, an ultrasound probe for transesophageal echocardiography (TEE) was installed such that the viewing angle as well as the distance to the aortic root mimics a TEE examination. Then, a volumetric ultrasound image of the root was acquired. We used a *GE Vivid E95* ultrasound system with the *6VT-D* probe. The size of the image was 84 × 202 × 84 with a voxel size of 0.71 × 0.49 × 0.71*mm*.

From this ultrasound volume, we cropped out a subvolume that shows the aortic root. Therefore, we manually identified the commissure plane, i.e., the horizontal slice through the aortic root that shows all three commissure points, i.e., the highest point of the commissures. We defined this plane as the uppermost slice of the subvolume and added the 31 layers below to it. Hence, the subvolume covered 22.72*mm* of the aortic root's height. Keeping this dimension fixed, we cropped the other two dimensions, i.e., horizontal slice images through the aortic root, to a size of 128 × 128 such that the aortic root was roughly in the image center of the slice images. We performed zero padding around the cropped root, i.e., areas of the new volume where no image information was available were filled with a gray value of zero. After rearrangement of the dimensions for convenience, the final image size was 32 × 128 × 128.

To get rid of imaging artifacts and background noise, we applied thresholding, setting all gray values smaller or equal to *t* = 80 to 0. Finally, the ultrasound images were scaled with the factor $\frac{1}{255}$ to lie in the range [0, 1].

#### 3.1.2. Leaflet Imaging

After the acquisition of ultrasound images of the aortic root, the three leaflets were extracted and assessed with high resolution, following the method of Hagenah et al. (23). Thus, the root was opened by a vertical cut in between the right-coronary and the non-coronary sinus. Then, the single leaflets were cut off the root wall and spread on a diffusive glass plate. Special care was taken to preserve the leaflet's original shape during extraction and spreading. From below the plate, blue illumination (470*nm*) was applied as this wavelength is absorbed in the collagen structures of the leaflets, leading to a high contrast. Then, a photograph was taken from above using a *Canon DS 6041* SLR camera with a fixed exposure time of $\frac{1}{6}s$. The resolution of this image was $0.037\frac{mm}{pixel}$.

**Figure 2:**
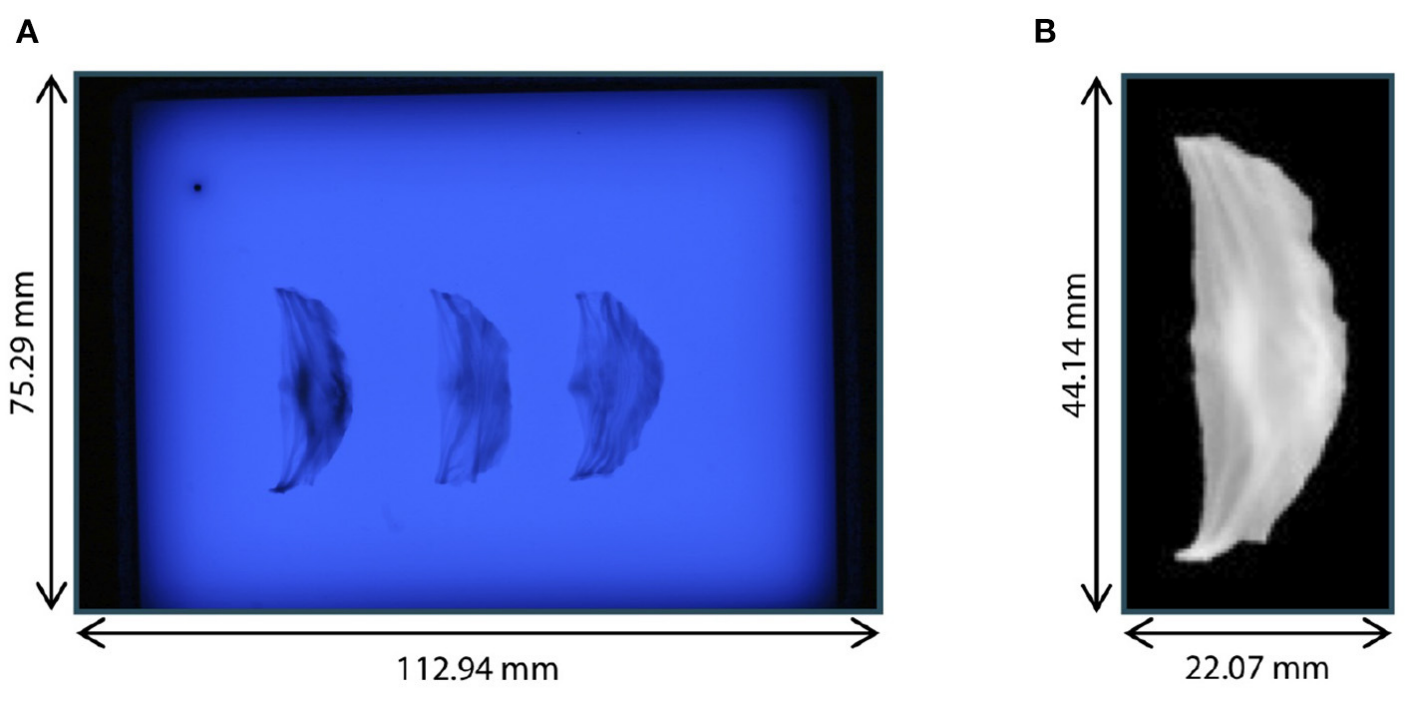
Leaflet image acquisition. **(A)** Raw photograph of illuminated plate with right-coronary, left-coronary, and non-coronary leaflet of one valve. **(B)** Right-coronary leaflet after preprocessing.

The preprocessing of the leaflets also followed (23). The individual leaflets were cropped out and the images were transformed to grayscale (range: [0, 255]) and inverted. All background pixels were set to 0 using thresholding with manually selected, individual thresholds for each leaflet to avoid holes in the segmentation. All thresholds were in the range from 158 to 168. The leaflets were centered in the image by translating the center of mass into the image's mid point. Then, the leaflet was aligned by rotating it around the image center so that the commissure points were vertically aligned. The resulting images were downsampled to a size of 128 × 64 pixels, leading to a resolution of $0.34\frac{mm}{pixel}$, and the grayscale values were scaled by the factor $\frac{1}{255}$ to lie in the range [0, 1]. Figure 2 shows an example raw image from the dataset as well as one preprocessed leaflet. In the scope of this study, we also made use of the leaflet image dataset presented in Hagenah et al. (23), containing 168 images of leaflets from 56 valves. Note that for these images, no corresponding ultrasound volume is known and they are used as an auxiliary dataset to reliably cover the full variety of leaflet shapes.

**Figure 3:**
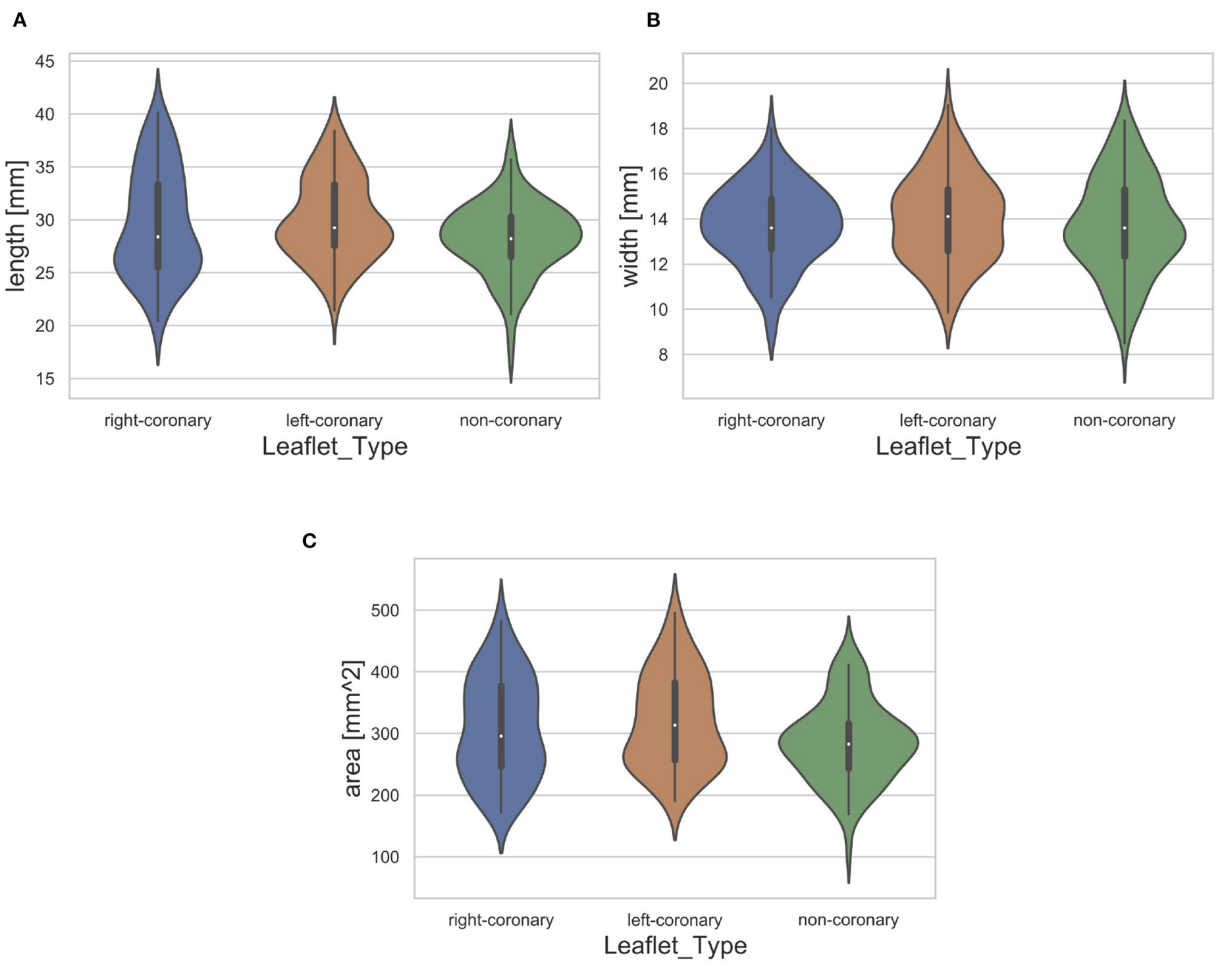
Distribution of leaflet length **(A)**, width **(B)**, and area **(C)** with regard to the leaflet type. The white dot represents the median value.

Furthermore, we assessed the distribution of leaflet shapes in the collected dataset. Therefore, we identified the length, width and area of each leaflet automatically. The length was measured as the maximal leaflet spreading among all image columns and the width as the maximum leaflet spreading among all image rows. The area was assessed as the number of pixels that show a part of the leaflet. The necessary segmentations were performed using thresholding (*t* = 0.45, corresponding to a grayscale value of 115). The resulting distribution of the dataset regarding the length, width and area for each leaflet type is visualized in Figure 3. Note that the distribution is visualized across all available leaflets, i.e., the data collected in this study as well as the auxiliary dataset. The high variance of leaflet shapes highlights the need for personalized modeling.

### 3.2. Problem Formulation

**Figure 4:**
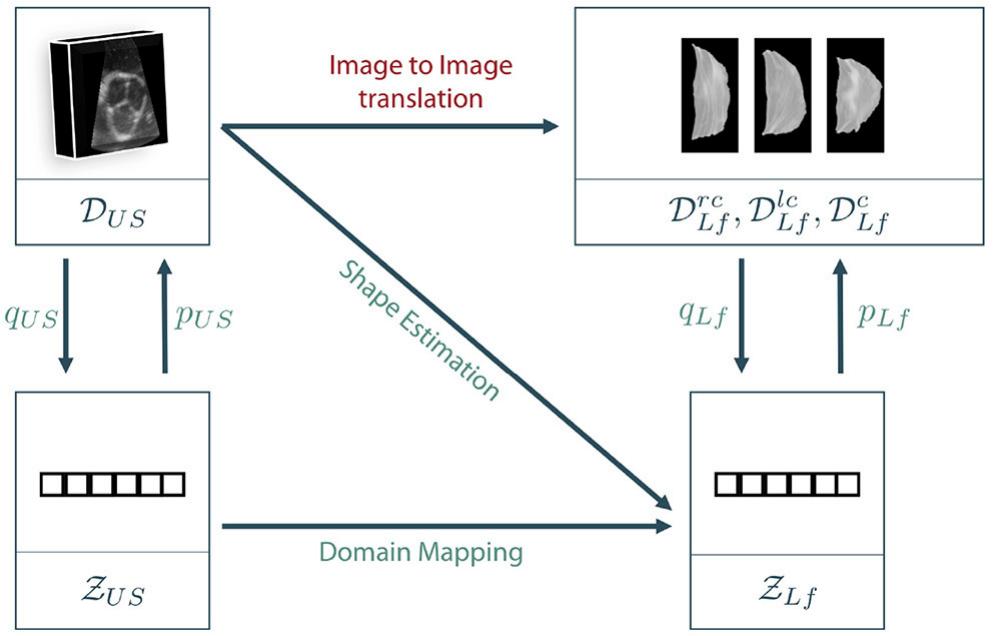
Sketch of our proposed approaches. As a direct mapping between the two data domains, also called *image to image translation*, is barely possible in the given scenario, we propose to encode the data to latent space descriptions using representation learning. Then, the latent leaflet description can be predicted based on the 3D ultrasound image, called *shape estimation*, or based on the latent description of the ultrasound volume, referred to as *domain mapping*.

As described in section 3.1, the data lies in two different spaces: The volumetric ultrasound data space $D_{US}\subseteq {\mathbb{R}}^{32\times 128\times 128}$ and the leaflet data spaces $D_{Lf}^{l}\subseteq {\mathbb{R}}^{128\times 64},l\in \left\{rc,lc,nc\right\}$ for the right-coronary, left-coronary and non-coronary leaflets, respectively. In addition, we define a latent representation Ƶ for each data space, leading to the latent spaces ${Ƶ}_{US}\subseteq {\mathbb{R}}^{m_{l}}$ and ${Ƶ}_{Lf}\subseteq {\mathbb{R}}^{n_{l}}$. Note that we assume a shared latent space representation for all three leaflet types, i.e., right-coronary, left-coronary and non-coronary. The latent space descriptions can be derived from the data space using representation learning on the corresponding dataset, respectively. Figure 4 shows the different coordinate spaces and their connection.

The general goal is to synthesize the unknown personalized leaflet images $I^{rc}\in D_{Lf}^{rc},I^{lc}\in D_{Lf}^{lc}$ and $I^{nc}\in D_{Lf}^{nc}$ of one patient based on the information contained in the patient's individual aortic root geometry given by $V\in D_{US}$. The direct mapping from *V* to *I*^*rc*^, *I*^*lc*^, and *I*^*nc*^ is known as *image-to-image translation* and typically requires a vast amount of training data as it is usually solved using GANs (see section 2). First encoding *V* to its latent representation *z*_*US*_ and then mapping to *I*^*rc*^, *I*^*lc*^, and *I*^*nc*^ is still interpreted as image-to-image translation as the only difference is an unsupervised pretraining of a subnetwork, which is a well-known technique in deep learning on small data (27). By encoding *I*^*rc*^, *I*^*lc*^, and *I*^*nc*^ to their latent representations $z_{Lf}^{rc},z_{Lf}^{lc},z_{Lf}^{nc}\in {Ƶ}_{Lf}$ using the encoder *q*_*Lf*_, the image to image translation problem can be reformulated as a regression problem where $z_{Lf}^{rc},z_{Lf}^{lc}$, and $z_{Lf}^{nc}$ should be estimated based on the ultrasound image data. If a decoder *p*_*Lf*_ is known, the corresponding shape leaflet images can be synthesized. Due to intense preprocessing of the leaflet image data, ${Ƶ}_{Lf}$ mainly contains shape information. Hence, predicting this latent information based on the volumetric ultrasound data *V* is referred to as *shape estimation*. Another option is to not only encode the leaflet images but also the ultrasound data. We assume that there is a connection between both latent spaces as they represent different parts and states of the same organ. However, the difference between both latent spaces is the domain gap between the data sets. One way to overcome this domain gap is to train a machine learning model to map from ${Ƶ}_{US}$ to ${Ƶ}_{Lf}$. Adding the encoder *q*_*US*_ and the decoder *p*_*Lf*_, a full path from an ultrasound volume to synthesized leaflet images is given. This approach is called *domain mapping*.

As described above, training an image-to-image translation model on very limited data until convergence is a challenging task. In addition, it is likely that an adversarial model learns the full distribution of leaflets without taking the prior information *V* into account, making the model fail in personalized shaping. Hence, we propose to follow the other two approaches, namely *shape estimation* and *domain mapping*. Details on our implementation of these approaches are given in the following sections.

### 3.3. Shape Estimation Approach

The goal of the *shape estimation* approach is to encode the leaflet shape, given as an image, into a latent space description and predict the individually optimal latent shape based on a 3D US image of the aortic root. Thus, the approach consists of two models: the leaflet autoencoder for shape encoding and the regression network for mapping from the US image to the latent representation.

For the leaflet autoencoder, we used a parameterized architecture (28). The encoder *q*_*Lf*_ consists of *n*_*c*_ convolutional layers with *n*_*f*_ filters, *ReLU* activation (29) and followed by a 2 × 2 average pooling each. Following this convolutional part, a flattening operation and fully connected layer (*ReLU* activation) with as many neurons as outputs of the last pooling layer connect to the bottleneck layer with *n*_*l*_ neurons, featuring *linear* activation. The decoder *p*_*Lf*_ follows the mirrored encoder architecture utilizing upconvolutisonal and upsampling. The autoencoder was trained using the *adam* optimizer, mean squared error loss and a batch size of 32 for 100 epochs (29).

It is assumed that there is a shared latent space description over all leaflet types, i.e., right-coronary, left-coronary and non-coronary leaflets. Hence, the autoencoder is trained on all three kinds of leaflets, leading to the dataset $D_{Lf}=D_{Lf}^{rc}\cup D_{Lf}^{lc}\cup D_{Lf}^{nc}$. To ensure a reliable representation, the data collected in Hagenah et al. (23) was added to the training data as an auxiliary dataset $D_{Lf}^{aux}$, containing 168 images of leaflets from 56 valves.

For the regression network, we propose to use a 3D convolutional neural network (CNN) with a VGG-like architecture (30). Thus, the network consists of *k*_*b*_ convolutional blocks. Each of these blocks consists of *k*_*c*_ 3D convolutional layers with *k*_*f*_ filters and *ReLU* activation. Each block is followed by a 2 × 2 average pooling layer. Behind the last convolutional block and a flattening operation, *k*_*d*_ fully connected layers with *ReLU* activation and *k*_*n*_ neurons each are attached, followed by the output layer with *k*_*l*_ neurons and *linear* activation.

We assume that the prediction of the individual shapes of the right-coronary, left-coronary, and non-coronary leaflets are independent. Hence, three regression models are created and trained, predicting one leaflet type each. The three models share the same architecture but are trained independently.

#### 3.3.1. Hyperparameter Analysis

To identify an optimal autoencoder architecture, i.e., an optimal combination of the hyperparameters *n*_*c*_, *n*_*f*_, and *n*_*l*_, we assessed multiple of these combinations regarding the model's ability to reconstruct the input image after propagating it through the model in a grid-search approach. Thus, we performed 10-fold Monte-Carlo crossvalidation (80% train, 20% test) on $D_{Lf}$ (28). After training the autoencoder on the training data, we propagated the test data through the model and compared the resulting reconstruction to the original image using root mean square error (RMSE). As mentioned before, the auxiliary dataset $D_{Lf}^{aux}$ was added to the training data within each fold. Table 1 shows all combinations of hyperparameters tested.

**Table 1:** Combinations of hyperparameters of the leaflet autoencoder assessed during hyperparameter analysis.

**Parameter**	**Values**
*n*_*c*_	**3**, 4, 5
*n*_*f*_	**16**, 32
*n*_*l*_	10, **20**, 30

*The optimal combination is marked in bold*.

After identifying an optimal autoencoder architecture, the hyperparameters of the regression network were optimized in a similar way. For multiple combinations of the hyperprameters *k*_*b*_, *k*_*c*_, *k*_*f*_, *k*_*d*_, and *k*_*n*_, the model's performance on predicting the latent shape representation of the corresponding leaflets for unseen ultrasound images is assessed using a 10-fold Monte Carlo crossvalidation (80% train, 20% test) on $D_{US}$ and $D_{Lf}$. Therefore, the autoencoder was trained on the training leaflet images using the optimal hyperparameters. Then, all training leaflet images were encoded to the latent space and the three regression networks were trained to predict the latent space representation of the respective leaflet based on the corresponding 3D ultrasound image. The accuracy was assessed by predicting the shape representation for the test US images, reconstruct images of the predicted leaflet shapes using the decoder and comparing these predicted images to the ground truth leaflet images, once again using RMSE. Table 2 shows all hyperparameter combinations assessed in this study.

**Table 2:** Combinations of hyperparameters of the regression networks assessed during hyperparameter analysis.

**Parameter**	**Values**
*k*_*b*_	3, **4**, 5
*k*_*c*_	1, 2, **3**
*k*_*f*_	16, **32**
*k*_*d*_	**1**, 2, 3
*k*_*n*_	50, **100**, 200

*The optimal combination is marked in bold*.

#### 3.3.2. Performance Analysis

After identifying optimal sets of hyperparameters for the autoencoder and the regression networks, we analyzed the performance of the shape estimation approach. Thus, we trained the autoencoder and the three regression networks on training data, using the respective optimal hyperparameters, and predicted the leaflet shapes for unseen test data. We did this using a 10-fold Monte Carlo crossvalidation (80% train, 20% test) on $D_{US}$ and $D_{Lf}$, while once again $D_{Lf}^{aux}$ was added to the training data for training the autoencoder. We compared the predicted leaflet images to their corresponding ground truth using four metrics: Jaccard similarity, Hausdorff distance, average symmetric contour distance (ASCD) and RMSE. The ASCD of two sets of contour points *X* and *Y* is given as


(1) $$\begin{array}{l} ASCD\left(X,Y\right)=\frac{ACD\left(X,Y\right)+ACD\left(Y,X\right)}{2} \end{array}$$

with the average contour distance (ACD) defined as


(2) $$ACD(X,Y)=\frac{\sum_{x\in X}\min_{y\in Y}d(x,y)}{\left\|X\right\|},$$

where *d*(*x, y*) is the euclidian distance between the points *x* and *y* (31).

To compute the Jaccard similarity, the Hausdorff distance and the ASCD, the leaflets were segmented in the predicted images utilizing thresholding (*t* = 0.45, corresponding to a grayscale value of 115).

### 3.4. Domain Mapping Approach

The key idea of the domain mapping approach is to encode both, the leaflet images as well as the volumetric US data, into a latent space description, respectively, and train a model to map from one latent space to the other. One important advantage is that the dimensionality of the mapping is much smaller than in the shape estimation approach. Hence, it is possible to learn this mapping using classical machine learning methods like random forests (RF) or multi-layer perceptrons (MLP) (28).

The autoencoder for the leaflet images is the same a presented in section 3.3. To find a representation of the US data, we propose a similar architecture but featuring 3D convolution and pooling. Hence, the encoder *q*_*US*_ consists of *m*_*c*_ 3D convolutional layers with *m*_*f*_ filters, *ReLU* activation and followed by a 2 × 2 × 2 average pooling each. Following this convolutional part, a flattening operation and fully connected layer (*ReLU* activation) with as many neurons as outputs of the last pooling layer connect to the bottleneck layer with *m*_*l*_ neurons, featuring *linear* activation. Once again, the decoder *p*_*US*_ follows the mirrored encoder architecture.

To find a mapping between the two latent spaces ${Ƶ}_{US}$ and ${Ƶ}_{Lf}$, we evaluated a random forest with *t* decision trees and an MLP with *l*_*h*_ hidden layers and *l*_*n*_ neurons in each hidden layer. For the MLP, all hidden layers used *ReLU* activation, while the output layer utilized *linear* activation.

#### 3.4.1. Hyperparameter Analysis

The optimal hyperparameters of the US autoencoder were identified in a similar way as for the leaflet autoencoder. Thus, we performed 10-fold Monte Carlo crossvalidation (80% train, 20% test) on $D_{US}$ and assessed the reconstruction accuracy on the test data using RMSE. Table 3 shows all evaluated combinations of hyperparameters.

**Table 3:** Combinations of hyperparameters of the ultrasound autoencoder assessed during hyperparameter analysis.

**Parameter**	**Values**
*m*_*c*_	**3**, 4, 5
*m*_*f*_	**16**, 32
*m*_*l*_	**20**, 100, 200

*The optimal combination is marked in bold*.

For finding the mapping between the latent spaces, we assessed different values of the hyperparameters for both learning methods. For the random forest approach, we analyzed the values 50, 100, 150, 200, 250 for *t*. For the MLP approach, we assessed the values 1, 2, 3, 4, 5 for *l*_*h*_ and 50, 100, 150, 200 for *l*_*n*_.

#### 3.4.2. Performance Analysis

After identifying optimal sets of hyperparameters, we evaluated the performance of the domain mapping approach on predicting the individual leaflet shapes based on a 3D US image on unseen data. Once again, we performed 10-fold Monte Carlo crossvalidation (80% train, 20% test) on $D_{US}$ and $D_{Lf}$. Thus, we trained both autoencoders on the training data (including $D_{Lf}^{aux}$ for the leaflet autoencoder) and encoded the training samples to their latent space representation. Then we trained models to predict *z*_*Lf*_ for a given *z*_*US*_. As described for the shape estimation approach, we propose to use three different models, one for each leaflet type, i.e., right-coronary, left-coronary and non-coronary. We evaluated RFs as well as MLPs for this regression, each of them using the optimal hyperparameters. After training the models, the test US images were encoded using *q*_*US*_, the latent leaflet representations were predicted and corresponding images were synthesized using the decoder *p*_*Lf*_. These predicted leaflet images were compared to the ground truth using Jaccard similarity, Hausdorff distance, ASCD and RMSE, once again with a thresholding if necessary (*t* = 0.45, corresponding to a grayscale value of 115).

## 4. Results and Discussion

At first, the results of the hyperparameter analysis are presented, followed by the performance analysis of both approaches. Afterwards, the results are discussed in detail and their impact on future research is given in an outlook paragraph.

### 4.1. Hyperparameter Analysis

As described above, all hyperparameters were optimized regarding a minimal RMSE. For both autoencoders, an architecture featuring three convolutional blocks with 16 filters each and a latent dimension size of 20 was identified as optimal, with an RMSE of 0.0617±0.0106 for the leaflet autoencoder and 0.0678±0.0200 for the ultrasound autoencoder.

For assessing the hyperparameters of the shape estimation approach, we used the optimal leaflet autoencoder hyperparameters and evaluated the parameter combinations of the CNN mapping from the ultrasound volume space $D_{US}$ to the latent space of the leaflet ${Ƶ}_{Lf}$. The optimal architecture features four convolutional blocks with three convolutional layers using 16 filters each, followed by a single fully connected layer with 100 neurons. This indicates that feature extraction needs a certain degree of abstraction, but the identified features are meaningful and can be processed with a simple classification model, i.e., a single fully connected layer. The RMSE between the true leaflet samples in the latent space description and the predicted ones reached with this architecture was 0.1331±0.0392.

For the domain mapping approach, we used the previously identified, optimal hyperparameters for both autoencoders and evaluated the hyperparameter influence on the model mapping from ${Ƶ}_{US}$ to ${Ƶ}_{leaflet}$. In the case of a Random Forest regression, 200 trees were found to perform best with an RMSE of 0.1365±0.0278, once again measured in the leaflet's latent space between the predicted and the true latent representation of the leaflet. Using an MLP for domain mapping, the optimal architecture consisted of four hidden layers with 100 neurons each, reaching an RMSE of 0.1386±0.0409. All hyperparameters that were identified to be optimal are marked in bold within Tables 1–3.

### 4.2. Latent Space Exploration

**Figure 5:**
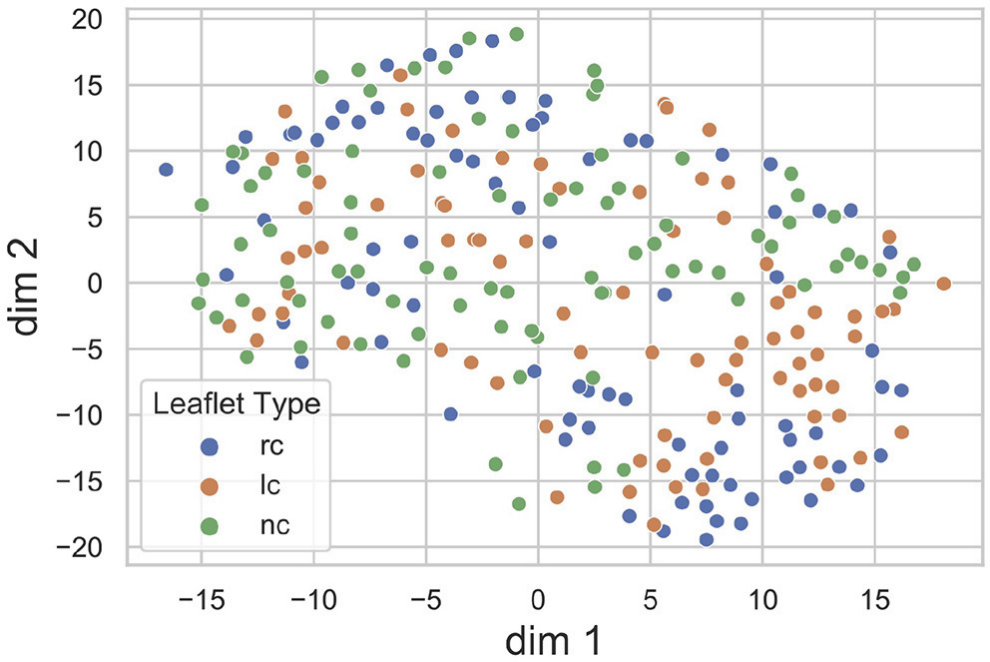
t-SNE embedding of the leaflet's latent space with the encoded dataset, divided into right-coronary (rc), left-coronary (lc), and non-coronary (nc) leaflets. There is a substantial overlap between the shape representations of the three leaflet types.

The proposed method heavily relies on the identification of a sufficiently accurate representation of the leaflet shape. Therefore, we performed an explorative analysis of the consistency and smoothness of the leaflet autoencoder's latent space. Figure 5 visualizes the distribution of leaflet shapes in the latent space as a t-SNE embedding (32). There is a substantial overlap between the shape representation of right-coronary, left-coronary and non-coronary leaflets. This finding validates our approach of identifying a unified representation for all leaflets $D_{Lf}$ instead of distinct representations for each leaflet type.

**Figure 6:**
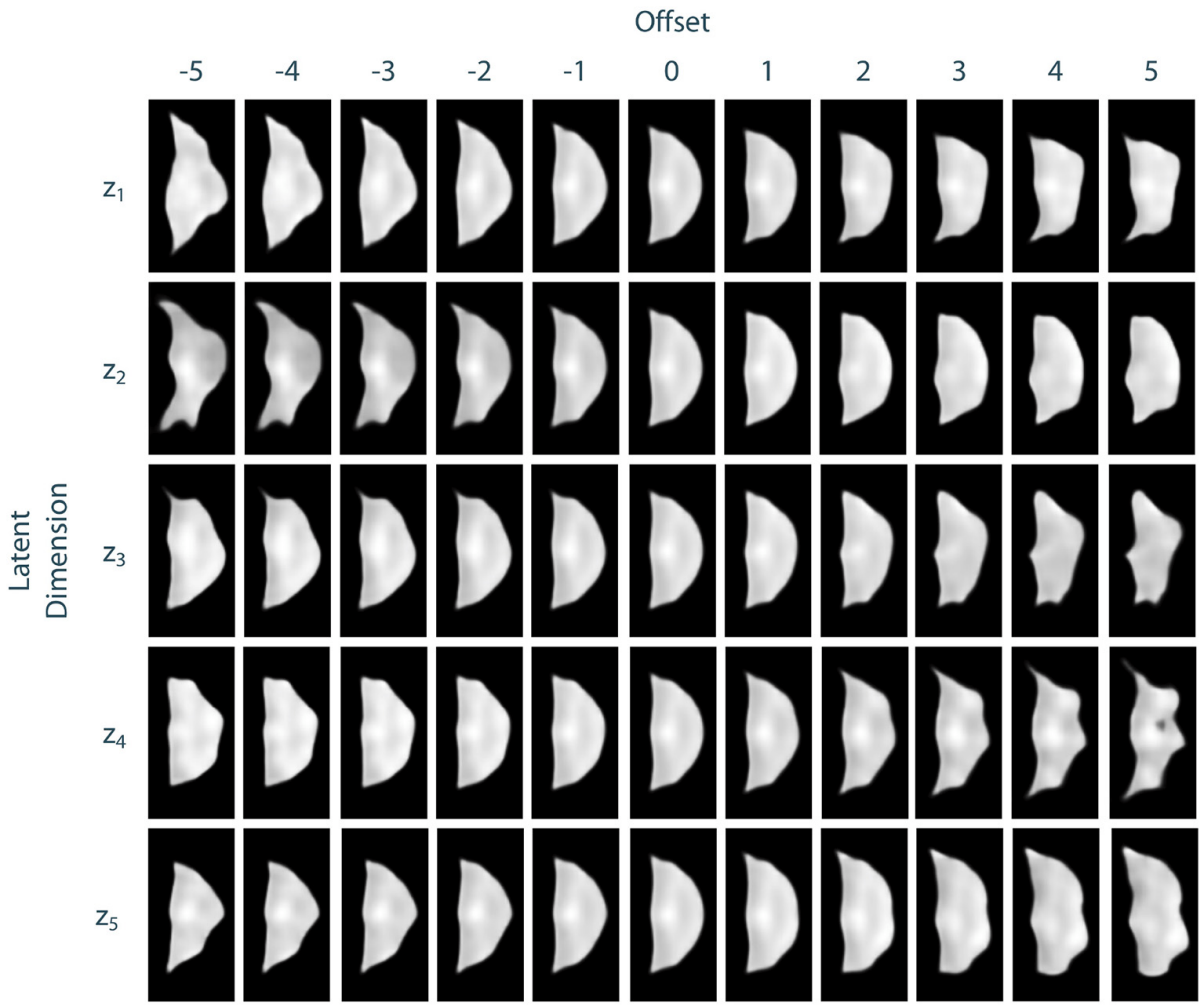
Exploration of the leaflet autoencoder's latent space. The median shape was manipulated by adding an offset to a specific dimension. Then, an image of this manipulated shape was synthesized. The resulting images are shown exemplarily for the first five dimensions of the latent space. Besides few outliers for extreme offsets, all shapes are realistic. The influences of the individual dimensions on the leaflet shape are consistent and complementary and the shape changes are smooth.

To assess the identified leaflet representation regarding its consistency and smoothness, we performed an exploration study in the latent space of the leaflet autoencoder. Therefore, after training the model, we encoded all leaflets from the dataset to the latent space and computed the median shape over all samples. Then, we manipulated the individual dimensions of this median shape by adding an offset *o* ∈ {−5, −4, −3, −2, −1, 1, 2, 3, 4, 5} to its value in the current dimension. Then, an image of the resulting manipulated shape was synthesized using the decoder network. Thus, the influence of the individual dimensions of the latent space, i.e., the identified abstract features, can be visualized and assessed. We found that most of the synthesized shapes were realistic with a small number of outliers for large offsets. Furthermore, the influence of the dimensions on the leaflet shape were consistent and complementary while the shape changes were smooth. Figure 6 exemplarily shows the synthesized images for the first five dimensions of the latent space. Based on these results, we assume that the leaflet representation is sufficiently consistent and smooth to use it for personalized shape synthesis.

### 4.3. Performance Analysis

**Figure 7:**
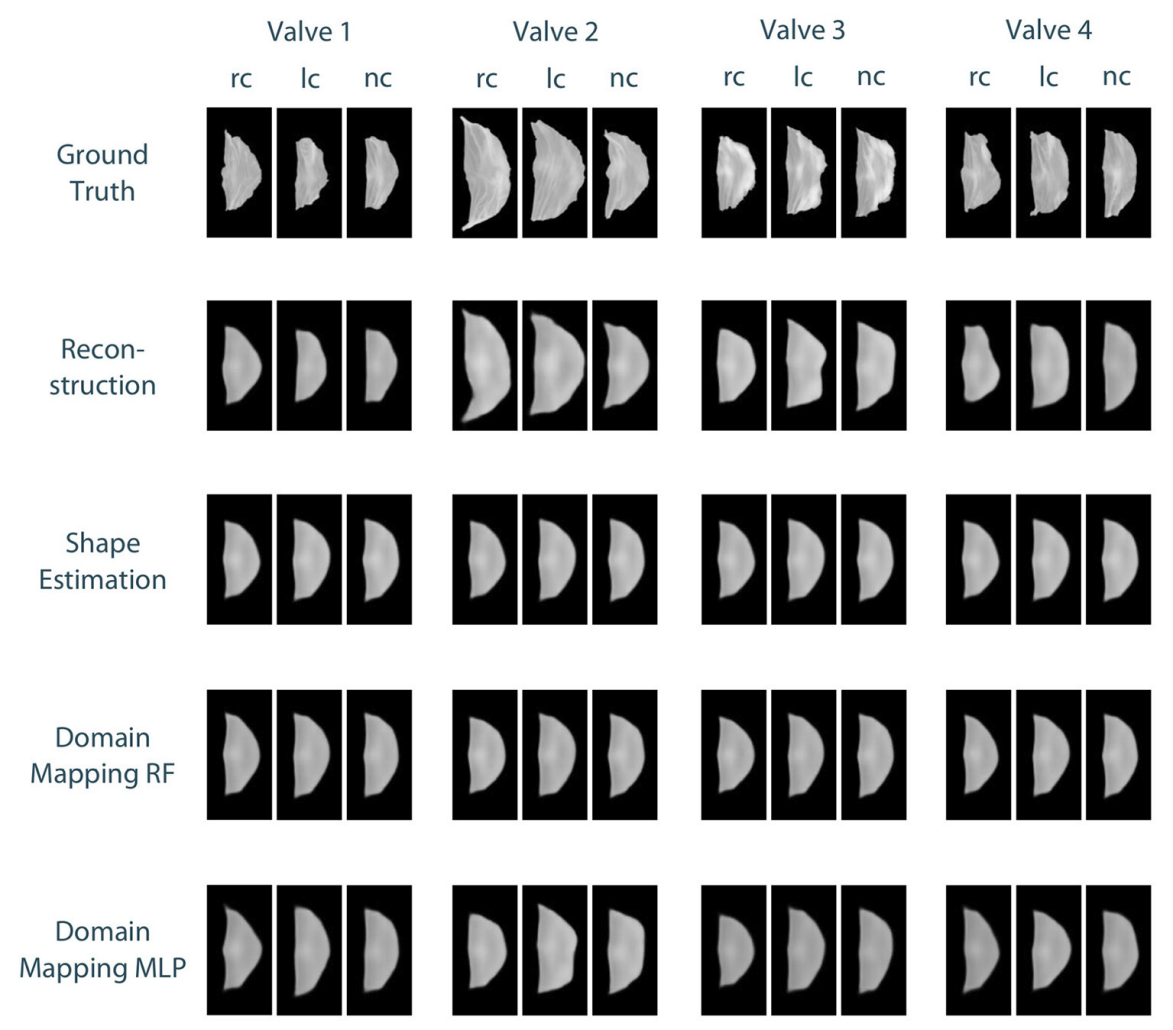
Qualitative results for leaflet shape synthesis with a geometric prior, exemplarily given for four valves (columns) with their right-coronary (rc), left-coronary (lc), and non-coronary (nc) leaflet, respectively. The rows show the ground truth, the reconstruction of the ground truth after propagating it through the whole autoencoder, as well as the synthetic leaflets produced by the different approaches, i.e., shape estimation, domain mapping with random forests, and domain mapping with multilayer perceptrons. All four valves were drawn from the test set of one fold of the crossvalidation.

**Figure 8:**
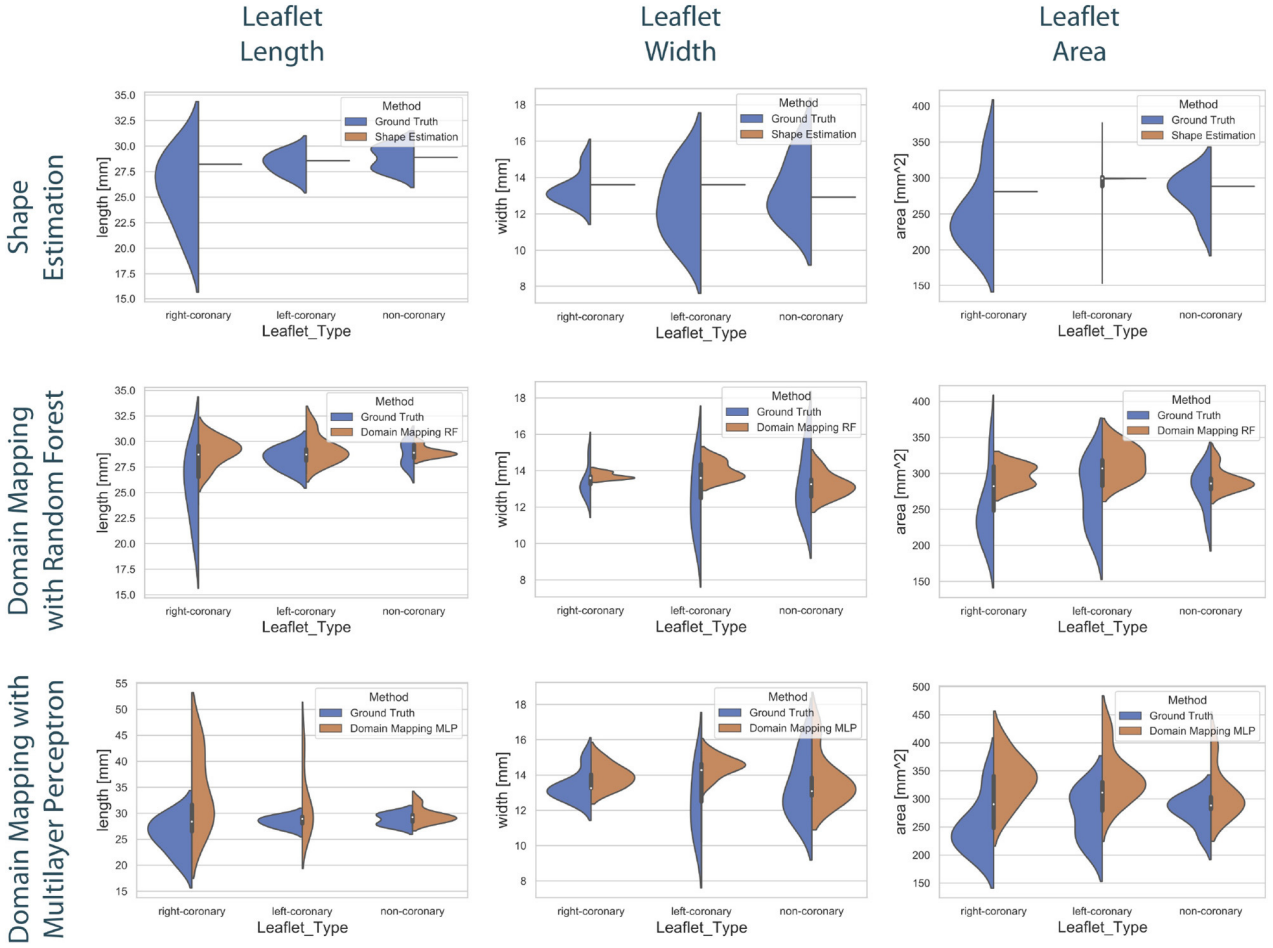
Distribution of the predicted leaflet shapes in comparison to the true distribution of the dataset $D_{Lf}$. The columns show the distribution of the length **(left)**, width **(middle)**, and area **(right)** in dependency of the leaflet type for the true distribution given in the dataset (blue) and the distribution in the predicted test set (orange). The rows correspond to the utilized prediction model, i.e., shape estimation **(top)**, domain mapping with RF **(middle)**, and domain mapping with MLP **(bottom)**. While shape estimation fails in predicting a variety of shapes, the MLP-based domain mapping approach outperforms the RF-based one regarding the coverage of the full distribution of observed leaflet shapes.

To compare the different approaches for leaflet shape synthesis, we evaluated all of them in a 10-fold crossvalidation. Figure 7 shows qualitative synthesis results exemplarily for four valves drawn from a test set within one fold of the crossvalidation. In general, all three synthesis methods are capable of providing realistic leaflet shapes in all cases. The shape estimation approach shows vanishing inter-patient variance and tends to predict the same leaflet for each individual. This is supported by the shape variance of the predicted leaflets regarding their length, width, and area compared to the ground truth distribution in the full dataset (see Figure 8). While the domain mapping approach with RF provides a fair variance, domain mapping with MLP shows the highest variance and coverage of the true shape distribution, highlighting its individualization performance. The latter approach is capable of following individual shapes relatively accurately in some cases, e.g., the right-coronary leaflet of valve 1, while it tends to overestimate the leaflet length. All presented methods struggle in predicting atypical leaflets, e.g., the right-coronary leaflet of valve 2.

**Figure 9:**
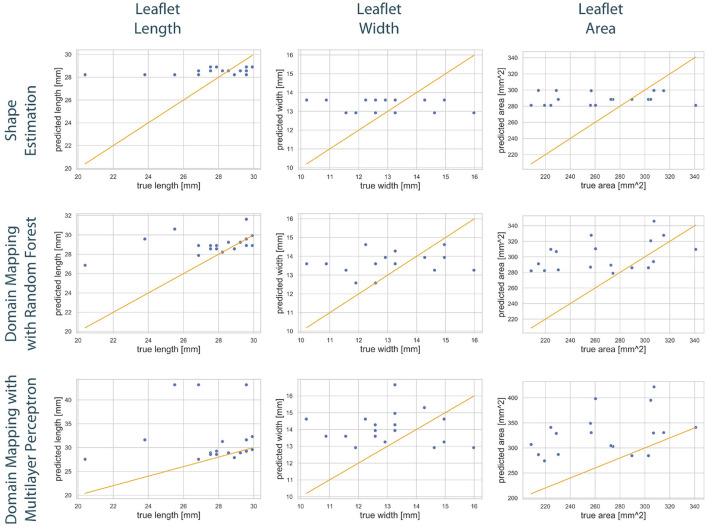
Correlation between geometric features of the predicted leaflets and the corresponding ground truth. The columns show the predicted length **(left)**, width **(middle)**, and area **(right)** in dependency of the true one, respectively. The assessed data points from the test set of one fold (blue) are scattered around the line sketching optimal correlation (orange). The rows correspond to the utilized prediction model, i.e., shape estimation **(top)**, domain mapping with RF **(middle)**, and domain mapping with MLP **(bottom)**. While shape estimation only predicts a limited number of possible outputs, both domain mapping approaches approximate the leaflet length sufficiently, with some severe outliers in the MLP-based approach. Both methods tend to overestimate the leaflet size. Please not the different scaling of the y-axes.

Figure 9 shows the correlation between the length, width, and area of the predicted leaflets and the corresponding ground truth for each prediction method. It is clearly visible that the shape estimation approach predicts a very limited number of possible shapes and therefor does not provide satisfying results. In contrast, both domain mapping approaches are capable of predicting the leaflet length well, with some severe outliers in the MLP-based approach. This highlights the necessity of encoding the ultrasound images to a latent representation and visualizes the higher robustness of the RF-based approach compared to utilizing MLPs. Both domain-mapping approaches tend to overestimate the size of smaller leaflets.

**Figure 10:**
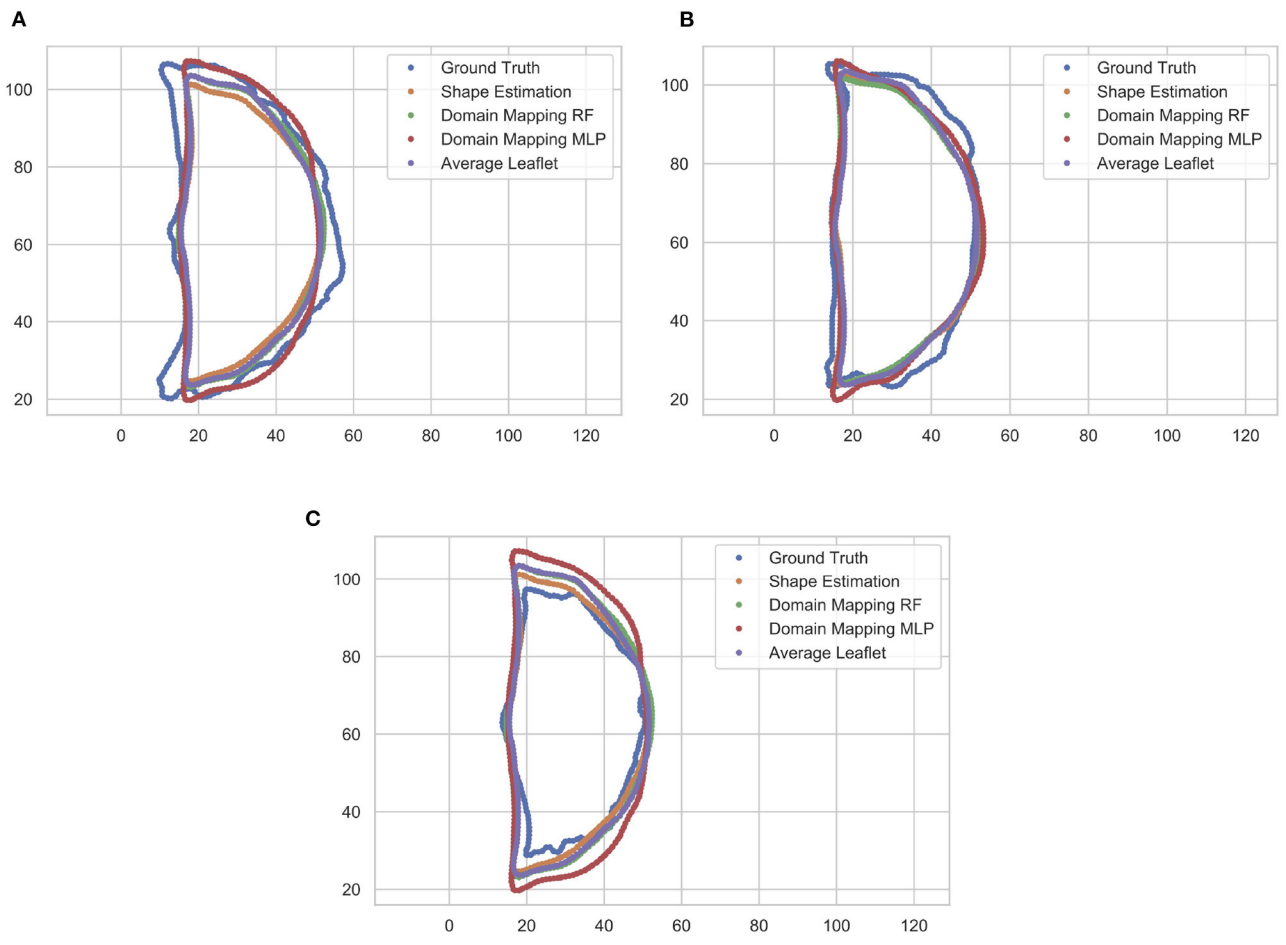
Comparison of predicted leaflet contour lines for the different prediction models as well as the ground truth, exemplarily shown for three typical cases. Typically, the domain mapping approach with MLP shows the best approximation **(A)**. For leaflets that are close to the average shape, all models provide a satisfying prediction **(B)**. If the leaflet is small, all prediction models tend to overestimate the size of the leaflet **(C)**. The contour lines are given in pixel coordinates.

These findings are supported by a qualitative assessment of the leaflet contour line prediction across all different models. Figure 10 exemplarily shows the comparison of predicted contour lines, together with the ground truth, for three typical cases. If the leaflet's shape is close to the average one, all models provide a satisfying contour line prediction. For leaflets that differ from the average shape, the domain mapping approach with MLP typically shows the best prediction regarding the contour line. However, for small leaflets, all prediction models tend to overestimate the size of the leaflet.

Table 4 shows the results of this study regarding Jaccard similarity, Hausdorff distance, ASCD and RMSE, averaged over all folds and all leaflet types. All three methods are capable of achieving a Jaccard similarity of more than 80%, while the *shape estimation* approach reaches the maximal value of around 83% coverage. This is most likely due to the high overlap between the test leaflets and an average one. However, regarding the contour line accuracy, the *domain mapping* approach provides lower ASCD values, indicating that it outperforms the *shape estimation* approach regarding finer details of the leaflet shape. As the image resolution was $0.34\frac{mm}{pixel}$, the *domain mapping* with an MLP achieves an ASCD of 0.97*mm* and hence is shown to predict the leaflet contour with sub-millimeter accuracy, with a maximum distance, i.e., Hausdorff distance, of 2.39*mm*.

**Table 4:** Accuracy of leaflet shape prediction for all methods, regarding RMSE, Jaccard similarity, Hausdorff distance, and ASCD.

**Method**	**RMSE**	**Jaccard**	**Hausdorff**	**ASCD**
Reconstruction of test data	0.0641 ± 0.0138	0.9463 ± 0.0123	2.86 ± 1.31	1.70 ± 7.53
Average leaflet	0.1440 ± 0.0449	0.8225 ± 0.0723	6.51 ± 3.99	3.19 ± 7.36
Shape estimation	**0.1383 ±0.0.0434**	**0.8310 ±0.0679**	**6.10 ±3.39**	4.03 ± 9.87
Domain mapping with RF	0.1417 ± 0.0479	0.8230 ± 0.0737	6.45 ± 4.29	3.10 ± 6.81
Domain mapping with MLP	0.1482 ± 0.0482	0.8060 ± 0.0796	7.02 ± 4.46	**2.84 ±5.01**

*The values for the Hausdorff distance and the ASCD are given in pixels, while the resolution was $0.34\frac{mm}{pixel}$. The table shows the mean value over all cross validation folds, averaged over all three leaflet types, and the standard deviation. As a comparison, the reconstruction accuracy of the test leaflets after propagation through the autoencoder is given, along with the prediction accuracy if the prediction is always the average leaflet shape*.

**Figure 11:**
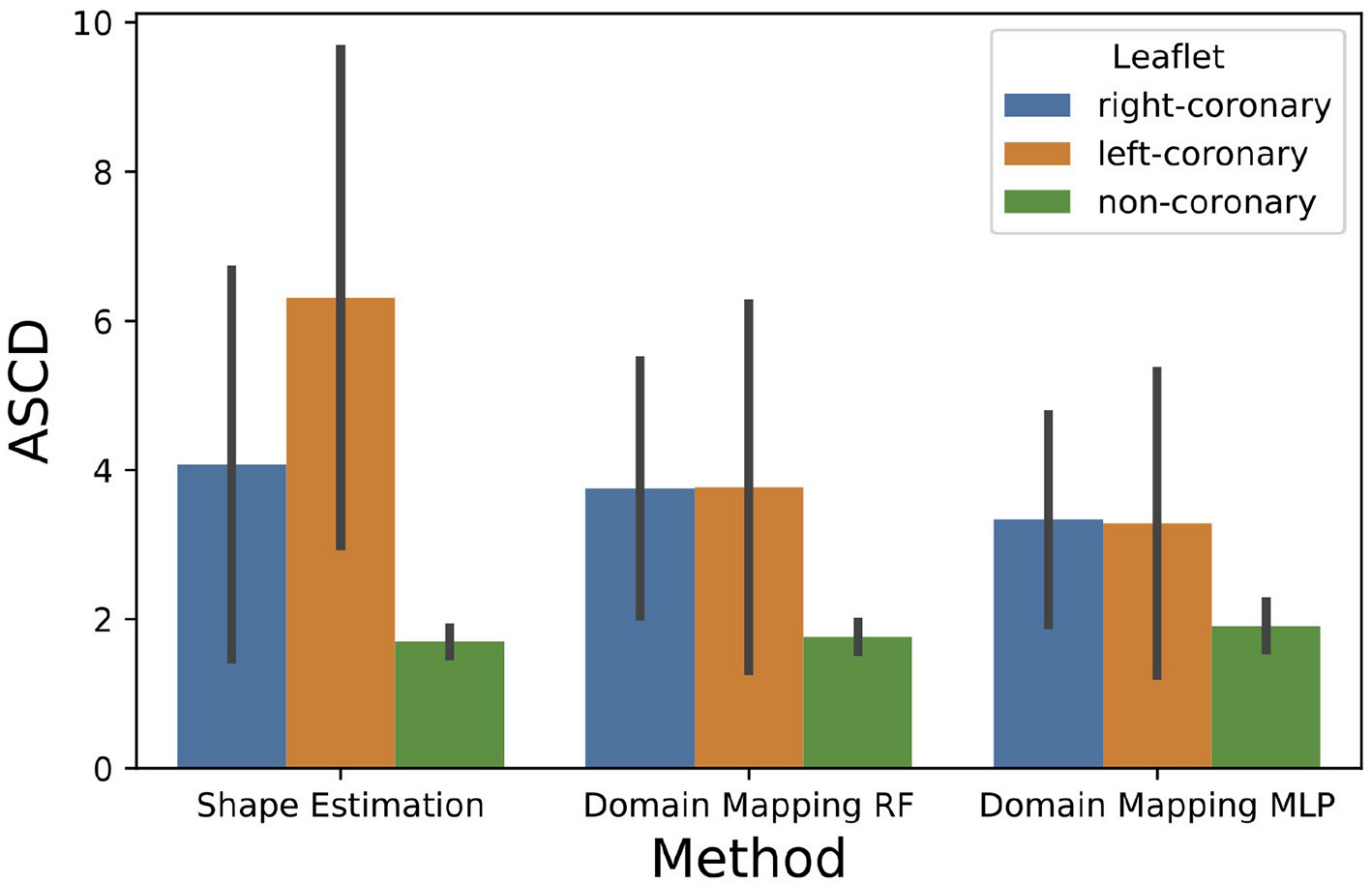
Accuracy of all methods regarding the predicted leaflet type, given as the ASCD (mean ± standard deviation) in pixels.

In Figure 11, the ASCD is given for each method regarding the accuracy in predicting the different leaflet types, i.e., right-coronary, left-coronary, and non-coronary. The results show that all methods are by far better in predicting the non-coronary leaflet shape than the other two leaflet shapes. This might be due to the fact that the inter-patient shape variance is higher for the right- and left-coronary leaflets than for the non-coronary one (see Figure 3). Additionally, the *shape estimation* performs worse in predicting the left-coronary leaflet than the right-coronary one, while both accuracy values are comparable in the case of *domain mapping*, regardless of the regression method. This indicates a higher robustness of the latter approach by utilizing meaningful features that describe the aortic root geometry that were identified during representation learning.

### 4.4. Discussion and Outlook

Our results indicate that bridging the high domain gap between volumetric ultrasound images of the aortic root and the planar shape of its three leaflets is possible even on a small amount of data. As the domain mapping with MLP provided personalized synthetic shapes, the ultrasound image carries enough information for this mapping when it is combined with a proper leaflet representation. However, the relevant information seems to be hidden within the ultrasound image and hard to extract as the shape estimation approach was not capable of providing adequately personalized synthetic images. By encoding the images to a latent space representation, it is possible to extract the relevant information, but still a relatively high level of abstraction is needed to make use of it as the optimal MLP featured four hidden layers. Accordingly, even though the RF grasps some parts of this information, its performance lags behind the MLP-based approach. Hence, in this scenario, the domain mapping approach with MLP clearly outperformed the other methods. However, this might be highly task-dependant and it is conceivable that the shape estimation method might perform better in tasks with lower-dimensional surrounding tissue data, i.e., 2D images. A broader analysis of the performance of the proposed methods for different applications and scenarios could provide important insights.

There are only few studies aiming at personalized aortic valve prosthesis shaping (33, 34). As the influence of personalized leaflet shaping on cardiac simulations or prosthesis performance has barely been studied, it is not clear what quantitative value for the accuracy of leaflet synthesis can be considered as clinically acceptable. The methods proposed in this work present the first model to personalize leaflet shapes in a data-driven way and hence open up possibilities for the asssessment of the influence of individual leaflet shaping, for example in a whole-heart simulation or, after fabricating a personalized prosthesis, in a left-heart simulator setup.

In this study, we only aimed on predicting the leaflets' shapes. However, regarding personalized biomechanical modeling or prosthesis development, a prediction of the leaflet's inner structure, i.e., the distribution of the prominent collagen fibers, would also be of high interest. The autoencoder used in this study smooths the inner structure and focuses on representing the general shape, which is desired in the scope of this work. However, if the leaflet representation could be extended to also contain information about the inner structure, the approaches described here would be capable of predicting the full leaflet structure, i.e., the shape and the inner structure. This once again highlights the flexibility of the presented framework.

It is important to note that in this study, all examined aortic roots and valves can be assumed to be healthy. In several clinical scenarios, this is not a limitation, e.g., for personalized aortic valve prosthesis shaping for patients suffering from aortic valve stenosis. In this case, the aortic root shape should not be altered significantly and hence, a prediction should still be possible. In contrast, there are clinical applications of the proposed framework that demand for predicting healthy leaflet shapes from pathological priors as well. This arises for patients suffering from morphological changes of the aortic root, e.g., due to an aneurysm. Therefore, the proposed framework should be evaluated in a setting where the geometric prior corresponds to a pathological state in the scope of a future study. As an alternative, it is possible to estimate the individual healthy aortic root shape based on the pathological one (35). The integration of this estimation step into the clinical pipeline might be a promising approach to apply the proposed framework also in scenarios where only a pathological prior can be assessed.

The output of the proposed method is a planar shape of the leaflet. Regarding the fabrication of personalized aortic valve prostheses, this is actually desirable since the planar shape can serve as a stencil to cut the leaflet out of the fabrication material. If the material mimics the leaflet's biomechanical properties sufficiently, the *in-vivo* shape under realistic prestrains will mimic the original healthy leaflets shape. However, it should be notes that in some application, the 3D curved shape of the leaflets under prestrains is desired, e.g., for when the fabrication material does not sufficiently reproduce the complex biomechanics or in digital twin scenarios. In these cases, a prediction of the planar, *ex-vivo* state without external strain is not sufficient. In contrast, Xu et al. (33) or Hsu et al. (34) aimed at directly modeling the 3D curved shape of the leaflet within the aortic root. But it should be noted that the presented framework is very flexible and can easily be adapted to other imaging modalities. Hence, if it is possible to collect image data of the leaflets *in-vivo* with a sufficient resolution, the framework could be used to predict this curved *in-vivo* shape as well. The only difference to the presented approach would be a different input for the leaflet autoencoder, the mapping approaches stay the same. This highlights the flexibility of the proposed framework and opens up new possibilities beyond personalized heart valve prosthesis shaping.

## 5. Conclusion

In this work, we presented a new framework to synthesize the shape of unknown anatomical structures based on the geometry of surrounding tissue by solving a domain mapping problem. We formalized the problem and proposed two general approaches to solve it. In an evaluation of this framework for the application of synthesizing aortic valve leaflet shapes based on volumetric ultrasound images of the aortic root, we could show that our method is capable of reliably synthesizing realistic leaflet shapes and that the geometric prior carries enough information to synthesize personalized leaflet shapes when both domains are encoded into a latent space and an MLP is used to learn a mapping between both latent spaces. This proof-of-concept study does not only open up plenty of applications of personalized aortic valve modeling but also presents a transferable approach for anatomical shape synthesis with geometric prior in general.

## Data Availability Statement

The datasets presented in this study can be found in online repositories. The names of the repository/repositories and accession number(s) can be found at: https://gitlab.rob.uni-luebeck.de/robPublic/avlid.

## Author Contributions

JH developed, implemented, and evaluated the presented methods. Furthermore, he contributed to the data collection and wrote most of the manuscript. MS contributed to the data collection, the experimental setups and workflows, and revised the manuscript. FE contributed to the general methodological framework as well as to the manuscript. Furthermore, he provided scientific advise during data collection and method implementation. All authors contributed to the article and approved the submitted version.

## Funding

This work was supported by the KI-LAB Lübeck [funded by the German Federal Ministry of Education and Research (BMBF), grant number 01IS19069] by providing access to an NVIDIA DGX A100, where all computations presented in this work were executed. Furthermore, this study was supported by the German Federal Ministry of Education and Research (grant number 13GW0228) by providing the ultrasound machine and probe. We acknowledge financial support by Land Schleswig-Holstein within the funding programme Open Access Publikationsfons.

## Conflict of Interest

The authors declare that the research was conducted in the absence of any commercial or financial relationships that could be construed as a potential conflict of interest.

## Publisher's Note

All claims expressed in this article are solely those of the authors and do not necessarily represent those of their affiliated organizations, or those of the publisher, the editors and the reviewers. Any product that may be evaluated in this article, or claim that may be made by its manufacturer, is not guaranteed or endorsed by the publisher.
